# Rehabilitación oral en síndrome de Sotos: reporte de caso y revisión exploratoria de la literatura

**DOI:** 10.21142/2523-2754-1402-2026-293

**Published:** 2026-04-04

**Authors:** Vicente Trabucco, Adriana Stuardo-Parada, Carolina Leiva

**Affiliations:** 1 Facultad Odontología, Universidad San Sebastián, Sede Valdivia. Valdivia, Chile. vtrabuccog@correo.uss.cl, adriana.stuardo@uss.cl, carolina.leiva@uss.cl Universidad San Sebastián Facultad Odontología Universidad San Sebastián Valdivia Chile vtrabuccog@correo.uss.cl adriana.stuardo@uss.cl carolina.leiva@uss.cl

**Keywords:** síndrome de Sotos, rehabilitación oral, prótesis parcial removible, odontología, Sotos syndrome, oral rehabilitation, dental prosthesis removable, dentistry

## Abstract

El síndrome de Sotos (SS) es un trastorno genético raro caracterizado por sobrecrecimiento, rasgos faciales distintivos y alteraciones dentofaciales. Aunque sus manifestaciones orales están bien descritas en la infancia, existe escasa evidencia sobre el manejo odontológico en adultos. Se describe, conforme a la pauta CARE, el caso de una mujer de 28 años diagnosticada con SS, quien presentaba características craneofaciales típicas del trastorno. La paciente consultó por desdentamiento parcial, por lo que se planificó una rehabilitación oral mediante prótesis parciales removibles. El plan de tratamiento consideró las limitaciones anatómicas, funcionales y cognitivas asociadas al síndrome, e incluyó una fase preventiva, adaptación protésica progresiva y controles clínicos periódicos. A los cuatro meses, se observó una adecuada adaptación protésica, estabilidad funcional y buena higiene oral. Se realizó una revisión exploratoria de la literatura (*scoping review*) siguiendo las directrices PRISMA-ScR. Se consultaron las bases de datos PubMed y Web of Science, identificando estudios que reportaran tratamientos odontológicos en pacientes con diagnóstico confirmado de SS. Se incluyeron seis reportes de casos publicados entre 2006 y 2023. Las manifestaciones orales más frecuentes fueron maloclusiones severas, hipodoncia, dientes supernumerarios y alteraciones esqueléticas. El manejo fue interdisciplinario, destacando terapias ortodóncicas en menores de edad y rehabilitaciones con implantes en adultos. La rehabilitación con prótesis removibles representa una alternativa válida y efectiva en adultos con SS, especialmente cuando existen limitaciones para tratamientos implantológicos. Este caso aporta nueva evidencia clínica y refuerza la necesidad de abordajes personalizados y seguimiento continuo en esta población.

## Abstract (en)

Sotos syndrome (SS) is a rare genetic disorder characterized by overgrowth, distinctive facial features, and dentofacial abnormalities. While its oral manifestations are well documented in childhood, there is limited evidence regarding dental management in adults. This case report, prepared in accordance with the CARE guidelines, describes a 28-year-old woman diagnosed with SS who presented with typical craniofacial features of the condition. The patient sought care for partial edentulism, and oral rehabilitation with removable partial dentures was planned. The treatment approach considered the anatomical, functional, and cognitive limitations associated with the syndrome, and included a preventive phase, progressive prosthetic adaptation, and periodic clinical follow-ups. After four months, the patient showed satisfactory prosthetic adaptation, functional stability, and good oral hygiene. A scoping review of the literature was conducted following PRISMA-ScR guidelines. Searches were performed in the PubMed and Web of Science databases to identify studies reporting dental treatments in patients with a confirmed diagnosis of SS. Six case reports published between 2006 and 2023 were included. The most frequently reported oral manifestations were severe malocclusions, hypodontia, supernumerary teeth, and skeletal abnormalities. Management was interdisciplinary, with a focus on orthodontic therapies in younger patients and implant-supported rehabilitations in adults. Removable prosthetic rehabilitation represents a valid and effective alternative for adults with SS, particularly when implant-based treatments are contraindicated. This case contributes new clinical evidence and highlights the importance of personalized approaches and continuous follow-up in this patient population.

## INTRODUCCIÓN

El síndrome de Sotos (SS), también denominado gigantismo cerebral, es un trastorno genético autosómico dominante descrito en 1964 y caracterizado por sobrecrecimiento, macrocefalia, rasgos faciales distintivos y retraso en el desarrollo psicomotor ^1^^-^^3^. En aproximadamente el 90 % de los casos la causa es una haploinsuficiencia del gen NSD1, localizado en el locus 5q35, mientras que en un porcentaje menor la etiología permanece incierta ^3^^,^^4^. Su prevalencia es baja, estimada en 1:15.000 nacidos vivos, y se han descrito diferencias genotípicas según la población: predominan las deleciones en pacientes japoneses y las mutaciones puntuales en poblaciones no japonesas ^5^.

Las características clínicas incluyen macrosomía, frente prominente, hipertelorismo, escoliosis e hipotonía congénita, además de complicaciones multisistémicas como cardiopatías, malformaciones genitourinarias, alteraciones oftálmicas y convulsiones ^3^^,^^5^. En la adultez, los rasgos faciales tienden a evolucionar hacia un rostro alargado, con predominio mandibular y mentoniano, junto a problemas crónicos como alteraciones dentarias, auditivas y musculoesqueléticas ^6^. Asimismo, son frecuentes las dificultades neuroconductuales, entre ellas retraso cognitivo, trastornos del lenguaje, ansiedad, signos del espectro autista y trastorno por déficit de atención e hiperactividad (TDAH), que condicionan el manejo clínico y odontológico ^7^.

En el ámbito orofacial, se han descrito múltiples anomalías dentarias y esqueléticas, tales como hipodoncia, oligodoncia, erupción prematura o ectópica, paladar ojival, mordida cruzada, mordida profunda y dientes supernumerarios, sin que se haya establecido un patrón oclusal característico ^5^^,^^8^^,^^9^. Estas alteraciones, junto con la hipotonía muscular, dificultan la planificación rehabilitadora, donde las opciones abarcan ortodoncia, cirugía ortognática y uso de implantes dentales cuando las condiciones óseas lo permiten ^5^.

Si bien las manifestaciones clínicas han sido ampliamente descritas en la infancia, la evidencia sobre su manejo odontológico en adultos es escasa. En este contexto, el presente artículo describe un caso clínico de rehabilitación oral en una paciente adulta con SS y complementando con una revisión exploratoria de la literatura para identificar las intervenciones dentales más frecuentemente reportadas en esta población.

## PRESENTACIÓN DEL CASO

### Este estudio siguió las pautas de reporte de casos (CARE)

**Figura 1:**
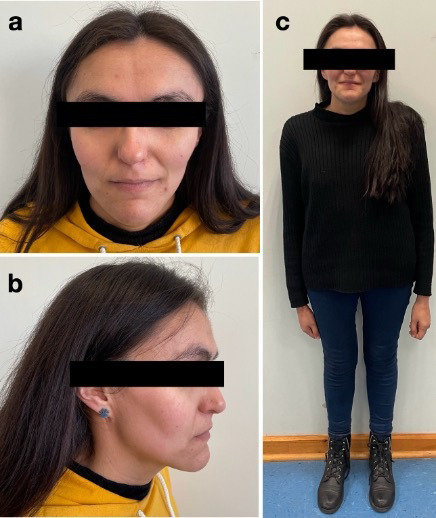
Fotografías extraorales que evidencian el fenotipo característico del SS en una paciente de 28 años. a) Macrocefalia, dolicocefalia y frente prominente. b) Mandíbula afilada. c) Escoliosis severa.

Paciente de sexo femenino, de 28 años, residente en Valdivia, Chile, con diagnóstico confirmado de SS, acudió al Centro de Salud de la Universidad San Sebastián por una urgencia odontológica que motivó la exodoncia de los molares 3.8 y 4.7. Posteriormente, fue derivada a la Clínica Integral del Adulto para una evaluación exhaustiva y planificación de la rehabilitación oral debido a su interés por recuperar sus dientes. Presenta fenotipo característico del síndrome: macrocefalia, frente prominente, mandíbula afilada, hipertelorismo, escoliosis severa (Fig. 1) y retraso cognitivo leve. Entre sus antecedentes quirúrgicos destacaban el cierre de comunicación interauricular, extirpación de un quiste ovárico y corrección quirúrgica de escoliosis.

**Figura 2:**
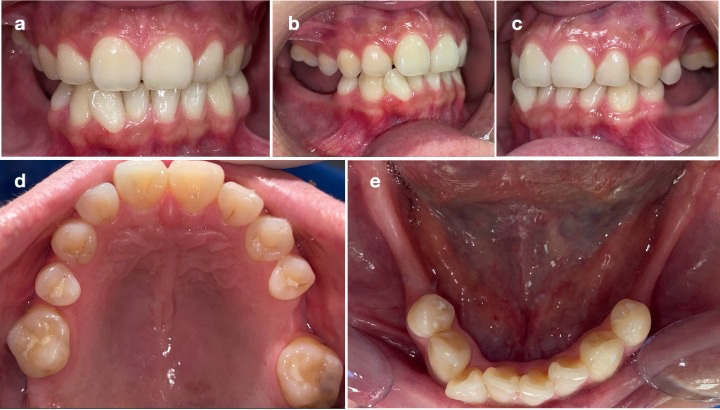
Fotografías intraorales que muestran los siguientes hallazgos: a) Desviación de la línea media. b) Relación canina clase III en el lado derecho. c) Relación canina clase I en el lado izquierdo. d) Arcada superior. e) Arcada inferior.

**Figura 3:**
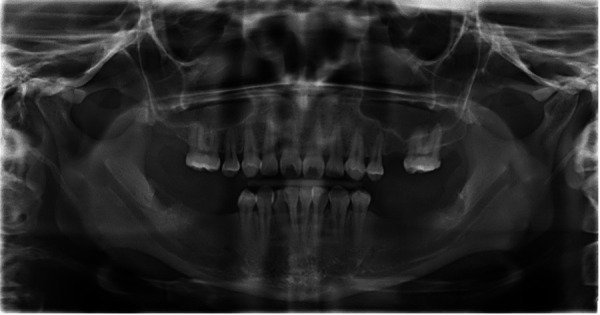
Radiografía panorámica inicial que muestra una asimetría en los cóndilos, neumatización de los senos maxilares y presencia de zonas de cicatrización ósea en las áreas edéntulas.

Al examen clínico se evidenció dolicocefalia, perfil facial recto, disminución del ángulo nasolabial y una apertura bucal de 49 mm sin signos de disfunción temporomandibular. Intraoralmente, presentaba paladar ojival, desviación de la línea media, desdentado parcial (ausencia de 1.7, 1.5, 2.5, 2.7, 3.7, 3.6, 3.5, 4.5, 4.6 y 4.7), relación canina clase III en el lado derecho y clase I en el izquierdo (Fig. 2), así como salud periodontal reducida con recesiones en el sextante V. La radiografía panorámica mostró asimetría condilar, neumatización de senos maxilares y áreas de cicatrización ósea en las zonas edéntulas (Fig. 3).

**Figura 4:**
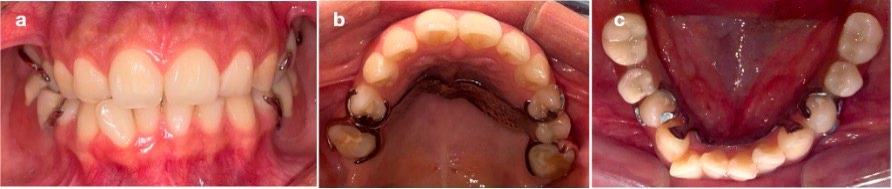
Fotografías intraorales del tratamiento rehabilitador con instalación de prótesis parciales removibles metálicas superior e inferior. a) Vista frontal. b) Arcada superior. c) Arcada inferior.

Se planificó un tratamiento rehabilitador en conjunto con la especialista en rehabilitación oral, considerando las condiciones anatómicas, funcionales y cognitivas de la paciente. El plan de tratamiento fue aprobado por la tutora legal de la paciente, quien firmó el consentimiento informado correspondiente. Tras la fase etiológica, que incluyó educación en higiene oral, técnica de cepillado modificada, barniz de flúor al 22.600 ppm, destartraje y pulido coronario, se procedió a la instalación de prótesis parciales removibles metálicas superior e inferior, con protocolo de adaptación progresiva (Fig. 4).

Se establecieron controles clínicos regulares para ajustar las prótesis, evaluar la adaptación funcional y mantener la salud periodontal, además de controles radiográficos semestrales para detectar lesiones incipientes.

A los cuatro meses de seguimiento, la paciente mostró buena evolución clínica, con estabilidad oclusal, ausencia de lesiones en la mucosa oral, higiene aceptable y sin signos de caries.

## REVISIÓN EXPLORATORIA DE LA LITERATURA

Se realizó una revisión exploratoria de la literatura (*scoping review*) empleando el protocolo PRISMA-ScR. El objetivo principal de esta revisión fue explorar la evidencia existente sobre las intervenciones odontológicas indicadas en pacientes con diagnóstico de SS.

**Figura 5:**
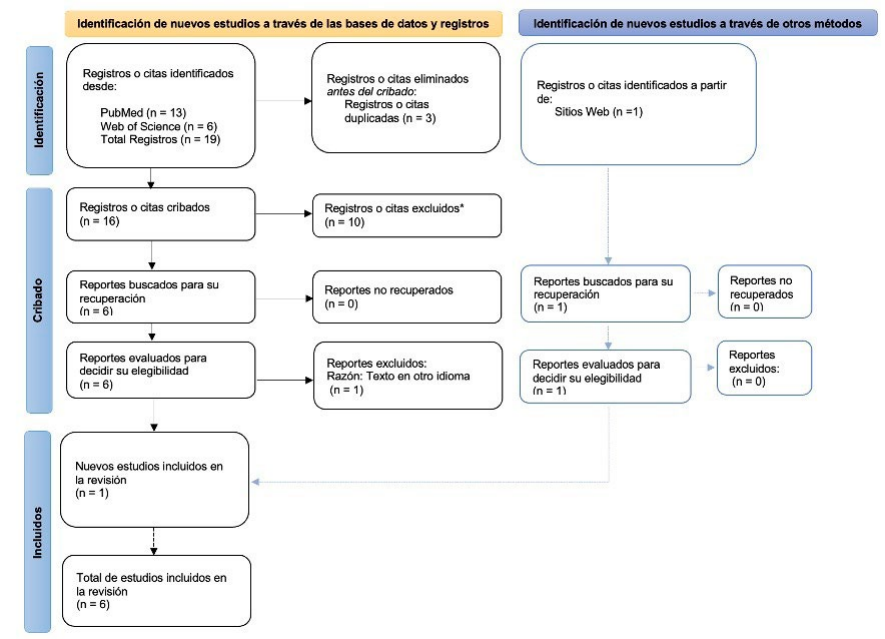
Diagrama de flujo PRISMA-ScR que representa el proceso de búsqueda y selección de los artículos finales.

Las bases de datos consultadas fueron: PubMed y Web of Science. Los términos seleccionados para la búsqueda fueron: Sotos syndrome AND (dental treatment OR oral treatment). Dos revisores analizaron de forma independiente los resultados obtenidos según los criterios de inclusión y exclusión, y un tercer revisor resolvió las discrepancias resultantes. Como criterios de inclusión, se consideraron reporte de casos o series de casos que incluyeran pacientes con SS y que hayan recibido tratamiento dental. Como criterios de exclusión, se descartaron aquellos estudios que proporcionen solo datos de prevalencia o que profundicen en el síndrome; estudios que no tengan relación con tratamientos dentales y estudios cuyo idioma no fuera el inglés ni el español. El proceso completo de selección se muestra en la Fig. 5.

## RESULTADOS

Se identificaron 6 reportes de casos clínicos publicados entre 2006 y 2023 que documentan tratamientos odontológicos en pacientes con diagnóstico confirmado de SS. Los detalles correspondientes a cada caso se resumen en la Tabla 1.

**Tabla 1:** Resumen de los casos clínicos incluidos en la revisión exploratoria sobre manifestaciones orales y tratamientos dentales en pacientes con SS.

Autor (año)	País	Características pacientes / Criterio diagnóstico	Manifestaciones orales	Tratamiento dental	Seguimiento
Gomes-Silva *et al*. (2006)	Brasil	Niño, 3 años. Con discapacidad intelectual leve	Prognatismo, paladar profundo, erupción dental prematura. Hipodoncia en incisivos mandibulares. *Dens evaginatus* y dientes fusionados (8.1-8.2)	Plan preventivo y rehabilitador: restauraciones de amalgama y restauraciones estéticas, ortodoncia (mantenedor de espacio)	3 años
Raitz y Laragnoit (2009)	Brasil	Niño, 11 años. Alta estatura, características faciales	Paladar profundo, Prognatismo mandibular, con apertura disminuida. Erupción dentaria prematura. 6 dientes supernumerarios: *Mesiodens* supernumerario	Extracción de supernumerarios, manejo multidisciplinario y controles odontológicos periódicos	3 años
Takano *et al*. (2012)	Japón	Adolescente, 17 años. Alta estatura, características faciales, retraso en el desarrollo	Clase II esquelética, arco maxilar estrecho, retrusión mandibular, mordida abierta anterior, mordida cruzada posterior, *overjet* de 14 mm, *overbite* de 11 mm	Expansión maxilar, distracción osteogénica mandibular, ortodoncia posterior	5 años poscirugía
Lago *et al*. (2019)	España	Hombre, 28 años. Alta estatura, características faciales, retraso en el desarrollo	Oligodoncia, concavidad marcada en la zona molar mandibular (fosa mandibular), arcada estrecha	Rehabilitación con implantes dentales (inicio en incisivos inferiores)	1 año (fase inicial)
Shioyasono *et al*. (2022)	Japón	Niño, 10 años. Discapacidad intelectual leve	Clase III esquelética, mordida cruzada anterior y posterior, desviación línea media, proclinación de incisivos superiores	Maxilar, mentonera y ortodoncia fija	1 año posretención
Oka *et al*. (2023)	Japón	Niño, 14 años. Discapacidad intelectual leve	Clase III esquelética, mordida cruzada posterior, paladar estrecho, persistencia de dientes temporales (todos los segundos molares temporales), oligodoncia	Expansión maxilar y tratamiento con aparatología fija, extracción de dientes deciduos (5.5-6.5)	40 meses

Los casos provienen de Japón (n = 3), Brasil (n = 2) y España (n = 1). De acuerdo con los datos presentados en la tabla, los pacientes correspondían al sexo masculino. Las edades variaron entre los 10 y 28 años, incluyendo cuatro pacientes pediátricos, un adolescente de 17 años y un adulto joven de 28 años. Los casos revisados revelaron que los pacientes diagnosticados con SS presentaron, de forma recurrente, alta estatura y rasgos faciales característicos (macrocefalia, frente prominente, mentón afilado). Además, en varios casos se documentaron discapacidad intelectual leve y retraso en el desarrollo.

Las manifestaciones orales del SS reportadas fueron amplias y heterogéneas, con predominio de anomalías esqueléticas y dentarias. Las maloclusiones severas estuvieron presentes en cinco de los seis casos, destacando relaciones clase III (n = 3), clase II (n = 1), mordida cruzada anterior y posterior (n = 3), mordida abierta (n = 1) y desviación de línea media (n = 1).

Entre las anomalías dentarias más frecuentes se encontró la hipodoncia y oligodoncia, la presencia de dientes supernumerarios, la erupción prematura de dientes temporales y permanentes, y la persistencia de piezas temporales en etapas avanzadas. Además, se documentaron patrones craneofaciales característicos como prognatismo, retrusión mandibular, paladar estrecho y alto, y concavidad en la región mandibular posterior.

El manejo odontológico de los pacientes fue interdisciplinario, adaptado a la edad del paciente y la complejidad del caso. En pacientes pediátricos y adolescentes, se emplearon estrategias ortodóncicas interceptivas como expansión maxilar, uso de mentonera, aparatología fija y distracción osteogénica mandibular ^1^^,^^5^^,^^9^^,^^10^.

En el caso del paciente adulto con oligodoncia, se planificó una rehabilitación con implantes dentales, iniciando con la zona anterior mandibular ^8^. Además, dos casos recibieron tratamientos preventivos y restauradores, y destacan la extracción de dientes supernumerarios y la implementación de programas de control de caries bajo supervisión continua ^1^^,^^2^.

El tiempo de seguimiento reportado en los casos clínicos revisados varió entre 12 meses y 5 años. En dos reportes se documentó un control prolongado de tres años ^1^^,^^2^, mientras que en otro se informó un seguimiento de cinco años posterior a la cirugía de distracción osteogénica ^10^. Un caso de rehabilitación implantosoportada indicó un año de control correspondiente a la fase inicial del tratamiento ^8^. En el manejo ortodóncico no quirúrgico se registró un año de seguimiento posterior a la fase de retención ^9^ y en la intervención ortodóncica con hipodoncia severa se consignaron 40 meses de observación clínica ^5^.

## DISCUSIÓN

El síndrome de Sotos (SS) es una condición de sobrecrecimiento caracterizada por rasgos craneofaciales distintivos, retraso del desarrollo y, en muchos casos, anomalías maxilofaciales y dentales. La literatura revisada confirma la alta variabilidad de las manifestaciones orales, así como la necesidad de abordajes odontológicos individualizados. Esta revisión incluyó seis casos clínicos publicados entre 2006 y 2023, en los que se evidencian patrones comunes como maloclusiones severas, hipodoncia y oligodoncia, dientes supernumerarios, paladar alto y erupción prematura ^1^^,^^2^^,^^5^^,^^8^^-^^10^.

Los tratamientos empleados en los casos pediátricos y adolescentes (edades entre 10 y 17 años) estuvieron centrados en terapias ortodóncicas interceptivas, como la expansión maxilar, uso de mentonera, distracción osteogénica y aparatología fija, lo que logró avances funcionales y estéticos a corto y mediano plazo ^5^^,^^9^^,^^10^. Estos abordajes tempranos permiten modular el crecimiento craneofacial y mejorar las condiciones para una rehabilitación posterior más conservadora. Sin embargo, su aplicación está supeditada a una detección precoz del síndrome y a una coordinación oportuna entre las especialidades médicas y odontológicas.

En cuanto al tratamiento restaurador en pacientes adultos, la literatura es escasa. El caso de Lago *et al*. (2019) ^8^ constituye la única referencia a una rehabilitación protésica fija mediante implantes dentales, la cual fue posible gracias a una adecuada disponibilidad ósea y ausencia de contraindicaciones sistémicas. Este caso pone de manifiesto que, aunque el tratamiento implantológico representa una alternativa viable, su indicación requiere una evaluación rigurosa que considere aspectos anatómicos, funcionales, cognitivos y económicos.

En este contexto, el presente estudio aporta un nuevo caso clínico de una mujer adulta joven diagnosticada con SS, cuya rehabilitación oral se efectuó mediante prótesis parcial removible superior e inferior. A diferencia del enfoque implantológico descrito por Lago *et al*. (2019) ^8^, en este caso se optó por una alternativa protésica removible debido a la ausencia de soporte óseo posterior adecuado, la preferencia expresada por la tutora legal y las limitaciones económicas asociadas al tratamiento. El tratamiento permitió restablecer la función masticatoria y mejorar la estética facial, evidenciando una adecuada adaptación de la paciente tras cuatro meses de seguimiento clínico.

Este caso amplía el espectro terapéutico reportado en la literatura y subraya la vigencia de las prótesis removibles como opción válida y efectiva, especialmente en pacientes con condiciones complejas que limitan otras alternativas. En un contexto clínico real, donde las condiciones ideales para rehabilitaciones implantosoportadas no siempre están presentes, la planificación debe orientarse a soluciones funcionales y personalizadas. Además, este caso refuerza la importancia de establecer protocolos de seguimiento sistemáticos, incluyendo controles periodontales, ajuste de las prótesis, aplicación de flúor y vigilancia radiográfica. Esto es especialmente relevante en pacientes con limitaciones cognitivas, quienes pueden enfrentar desafíos en el autocuidado oral y en la identificación oportuna de posibles complicaciones.

Por último, se destaca la necesidad de fortalecer la integración entre servicios médicos y odontológicos, así como la formación de equipos interdisciplinarios capaces de abordar de manera integral las necesidades de esta población. A pesar de su baja prevalencia, el SS plantea desafíos significativos en el ámbito odontológico, por lo que se requiere generar más evidencia clínica y fortalecer las redes de apoyo para la atención de personas con condiciones genéticas complejas.

## CONCLUSIONES

La rehabilitación oral en pacientes adultos con SS representa un desafío clínico poco explorado. Este caso demuestra que las prótesis removibles pueden ser una opción funcional y estética válida cuando existen limitaciones anatómicas, cognitivas o económicas. La revisión de la literatura refuerza la necesidad de abordajes personalizados y seguimiento continuo, así como de generar más evidencia que oriente el manejo odontológico integral en esta población.

## Financiamiento:

Investigación autofinanciada
